# Modulation in Elastic Properties of Upper Trapezius with Varying Neck Angle

**DOI:** 10.1155/2019/6048562

**Published:** 2019-03-03

**Authors:** Jun Zhang, Jiafeng Yu, Chunlong Liu, Chunzhi Tang, Zhijie Zhang

**Affiliations:** ^1^Clinical College of Acupuncture, Moxibustion, and Rehabilitation, Guangzhou University of Chinese Medicine, Guangzhou, China; ^2^Department of Rehabilitation Medicine, The First Affiliated Hospital of Zhengzhou University, Zhengzhou, China; ^3^Luoyang Orthopedic Hospital of Henan Province, Orthopedic Hospital of Henan Province, Luoyang, China

## Abstract

**Background:**

Neck and shoulder complaints caused by poor posture may influence upper trapezius stiffness. The relationship between the shear elastic modulus of the upper trapezius and cervical flexion angles is unknown. Therefore, it is essential to assess upper trapezius stiffness during cervical flexion. The objectives of this study were to (1) determine the intra- and interoperator reliabilities of evaluating upper trapezius stiffness and calculate the minimal detectable change (MDC); (2) examine the elastic modulus alterations of the upper trapezius during cervical flexion; and (3) explore the difference of upper trapezius stiffness between the dominant and nondominant sides.

**Methods:**

Twenty healthy male participants were recruited in this study. The shear modulus of the upper trapezius was evaluated by two independent investigators using shear wave elastography (SWE) during cervical flexion at 0° and 50°.

**Findings:**

The intraoperator (intraclass correlation coefficient (ICC) = 0.85–0.86) and interoperator (ICC = 0.94–0.98) reliabilities for measuring the shear elastic modulus of the upper trapezius during the cervical flexion ranged from good to excellent. An increase of 35.58% in upper trapezius stiffness was found at 0° to 50° of cervical flexion, and the MDC was 7.04 kPa. In addition, a significant difference was obtained in the elastic modulus of the upper trapezius muscle between the dominant and nondominant sides (*P* < 0.05).

**Conclusions:**

Our findings revealed that SWE could quantify the elastic modulus of the upper trapezius and monitor its changes. Therefore, further studies are required to delineate the modulation in upper trapezius muscle stiffness among subjects with neck and shoulder pain.

## 1. Introduction

Neck pain is a common complaint that seriously diminishes quality of life [1, 2]. The annual incidence of neck pain now exceeds 30% [3]. Additionally, neck pain is considered a more general consequence of musculoskeletal disorders in certain professions than lumbar and knee discomforts [4]. The upper trapezius muscle, which spans the neck and shoulder, contributes to normal cervical vertebra and scapula motion [5]. The biomechanical properties of the upper trapezius can be influenced among individuals with neck pain as evidenced by the significant increases in upper trapezius activity on electromyography (EMG) and stiffness by SWE [6–8]. Therefore, it is meaningful to evaluate the biomechanical properties of the upper trapezius to further explore its biomechanical mechanism and provide standard guidance in rehabilitation plans.

The upper trapezius muscles, as neck extensor muscles, play a vital part in maintaining cervical stability despite head and neck movement. Intensive electronic device users who assume a fixed posture for a long term are prone to developing exhaustion and neck and shoulder pain [9]. The activity of the upper trapezius was significantly associated with neck and elbow flexion angles while using the cellphones [10]. In addition, upper trapezius fatigue became more serious during neck flexion of 0° to 50° [11]. When the cervical muscles were flexed at the reextension phase, the EMG signal of the shoulder extensor muscles was sufficiently powerful [12]. Based on the findings of previous studies, the biomechanical properties of the upper trapezius were influenced by the cervical flexion angle.

The biomechanical properties of the upper trapezius muscle were recently quantified using various techniques. For example, EMG is still widely used to detect muscle conditions [11, 12]. However, there are some limitations, including the source of the EMG signals being complex and the results being particularly influenced by interference between physiology, muscle tissue, external environmental noise, and sweating [13]. Nevertheless, SWE as a novel technology used to assess various degrees skeletal muscle stiffness is increasingly common in scientific fields [14–16] and can overcome these limitations. The principle of SWE technology is based on different shear wave velocities (*V*) generated by pulses in various biological tissues [17]. Young's modulus (*E*), one shear modulus, is generally used to indirectly reflect tissue stiffness, namely, *E* = 3*ρv*^2^, in which *ρ* represents the tissue density. Excellent reliability and feasibility for assessing the deltoid, supraspinatus, and infraspinatus muscles using SWE in different positions (abduction, external rotation, and scaption) were reported [18]. During arm abduction, excellent intra- and interoperator reliabilities for the upper trapezius stiffness were seen as evidenced by ICC > 0.78 and a standard error of the mean (SEM) < 6.23 kPa [19]. Furthermore, SWE can be used to quantify individual neck extensor modulation during isometric contraction [20]. Therefore, SWE has the potential to estimate upper trapezius muscle stiffness. To our knowledge, no studies have examined the upper trapezius muscle stiffness modulation at different neck flexion angles.

The objectives of this study were to (1) determine the intra- and interoperator reliabilities of evaluating upper trapezius elasticity and calculate the minimal detectable change; (2) examine the elastic modulus alterations of the upper trapezius during cervical flexion; and (3) explore the difference of upper trapezius stiffness between the dominant and nondominant sides.

## 2. Methods

### 2.1. Ethical Approval

The study was approved by the Human Subject Ethics Committee of the Guangzhou University of Chinese Medicine. The experimental procedures were fully explained to each subject before the study. All subjects provided written informed consent prior to the experiment, and all of the study procedures adhered to the principles of the Declaration of Helsinki.

### 2.2. Subjects

Twenty healthy male subjects from Luoyang Orthopedic-Traumatological Hospital participated in this study. Age, height, weight, body mass index (BMI), and weekly exercise time of each subject were recorded. The subjects were prohibited from exercising for 48 h before the experiment. Subjects with a history of neck or shoulder pain, orthopedic disease, or upper-limb neuropathy were excluded.

### 2.3. Equipment

The shear modulus of the upper trapezius muscle was quantified using the SWE with an Aixplorer ultrasound unit (SuperSonic Imagine, Aix-en-Provence, France) equipped with a 40 mm linear ultrasound transducer (2–10 MHz). The musculoskeletal mode was used to estimate the shear modulus of the upper trapezius muscle with temporal averaging (persistence), penetration mode, and 85% opacity. The range of the color scale was adjusted from 0 to 200 kPa.

### 2.4. SWE for Measuring Upper Trapezius Muscle

**Figure 1:**
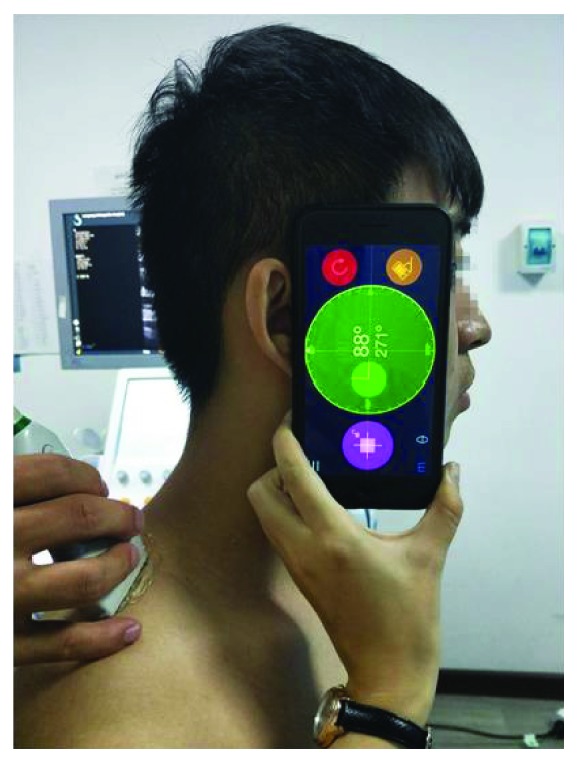
Participant position at 0° of cervical flexion with a Goniometer Pro assessing upper trapezius stiffness.

**Figure 2:**
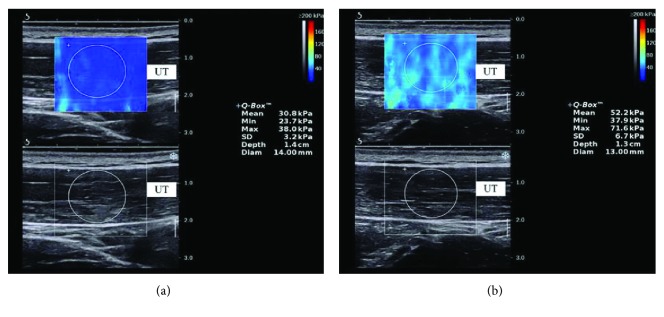
SWE maps of the upper trapezius muscle. Upper images: color-coded box presentations of the upper trapezius elasticity are shown in the upper image (the image color represents stiffness degree: red indicates stiff, while blue indicates soft). Lower images: B-mode images of the upper trapezius (UT: upper trapezius).

The SWE was used to quantify the upper trapezius muscle stiffness. The subjects sat on a chair with the shoulders in a neutral position and knees at 90° of flexion. Before testing, the participants were allowed to have a 5 min rest in a seated position. The cervical flexion angle was measured by a new iPhone application (Goniometer Pro) (Figure 1). Pourahmadi et al. measured cervical flexion angles using Goniometer Pro and reported good reliability: ICC > 0.65, SEM < 3.11°, and MDC < 8.62° [21]. In our study, 10 subjects were recruited to calculate Goniometer Pro reliability during 50° of cervical flexion: ICC > 0.71, SEM < 0.61°, and MDC < 1.96°. The measurement sites were marked with a marker at the midpoint between the seventh cervical spinous process and the acromion [22]. The marked site for each subject was cleaned after the experiment. Before the scanning, ultrasound gel was applied to the skin around the probe location. On the B-mode image, the probe was placed perpendicularly to the skin and slightly adjusted so it was parallel to the upper trapezius muscle fibers to obtain a clear image. Once an image without a muscle anisotropic artifact was determined, we switched to E mode to quantify the elastic modulus of the upper trapezius muscle (Figure 2). The size of the circular regions of interest (ROIs) was defined as the thickness of the upper trapezius [22]. The mean value of three measurements was used in this study.

The bilateral sides of the elastic modulus of the upper trapezius muscle were estimated using SWE. The dominant side was subjected to the intra- and interoperator reliability tests. To assess intraoperator reliability, all subjects were examined by operator A (ZJ) using SWE at 0° and 50° of neck flexion. The same subjects were evaluated again by operator A (ZJ) 5 days later. For the evaluation of interoperator reliability, all subjects were assessed by both operators (ZJ and ZJP) once at 30 min intervals. The operators were blinded to the measurement results during the test. After completing the measurement task at each angle, the participants were allowed to relax for 2 mins.

### 2.5. Statistical Analysis

SPSS version 19.0 software (SPSS Inc., Chicago, IL, USA) was used to perform the statistical analyses. The demographic information was calculated by descriptive statistics. A paired *t*-test was performed to compare the mean elastic moduli of the upper trapezius muscle between 0° and 50° of cervical flexion and verify the differences between the elastic moduli of the upper trapezius muscle between the dominant and nondominant sides. Intra- and interoperator reliabilities were determined by the calculation of the intraclass correlation coefficient (ICC) with 95% confidence interval. The intraoperator reliability was evaluated using the ICC (3,1) (two-way mixed-effect model, consistency), and the interoperator reliability was assessed using the ICC (2,2) (two-way random effects model, absolute agreement). The standard error of measurement (SEM) was computed by the formula $\text{SEM}=\text{standard deviation}\times \sqrt{1-\text{ICC}}$, while the MDC was calculated by the formula $\text{MDC}=1.96\times \text{SEM}\times \sqrt{2}$. Bland and Altman plots further intuitively indicated the degree of agreement for assessing intra- and interoperator reliabilities. All measurement data are expressed as mean (standard deviation), and values of *P* < 0.05 were considered statistically significant.

## 3. Results

### 3.1. Demographic Data

Demographic information including age, weight, height, BMI, and weekly exercise hours for all subjects are shown in Table 1. All subjects were able to complete the action of cervical flexion without discomfort under the guidance of the operators.

### 3.2. Intra- and Interoperator Reliabilities

The related statistical parameters for intra- and interoperator reliabilities for assessing upper trapezius muscle stiffness of the dominant shoulder are summarized in Table 2. The mean stiffness values of the upper trapezius were 40.47 kPa for operator A in test 1, 39.90 kPa for operator A in test 2, and 41.01 kPa for operator B during the cervical flexion at 0°. The mean stiffness values of the upper trapezius were 62.83 kPa for operator A in test 1, 64.12 kPa for operator A in test 2, and 63.20 kPa for operator B during the cervical flexion at 50°. The ICC values of intraoperator reliability were good in the context of cervical flexion at 0° (ICC = 0.86; 95% CI = 0.69–0.94; SEM < 2.59 kPa; and MDC < 7.20 kPa) and cervical flexion at 50° (ICC = 0.85; 95% CI = 0.67–0.94; SEM < 5.01 kPa; and MDC < 13.89 kPa). The ICC values of interoperator reliability were excellent in the context of 0° of cervical flexion (ICC = 0.98; 95% CI = 0.96–0.99; SEM < 2.69 kPa; and MDC < 7.48 kPa) and 50° of cervical flexion (ICC = 0.94; 95% CI = 0.86–0.97; SEM < 5.01 kPa; and MDC < 13.89 kPa).

**Figure 3:**
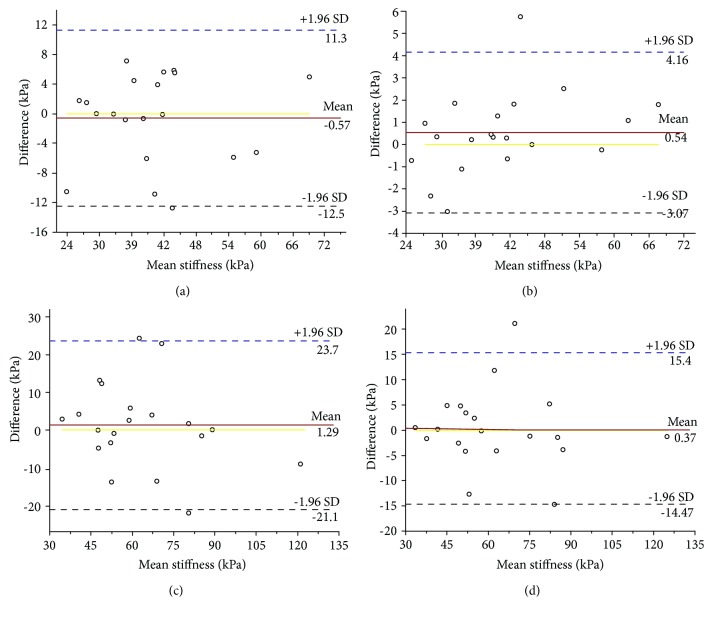
Bland-Altman plots of intra- and interoperator reliabilities for measuring the upper trapezius at 0° of cervical flexion. (a, b) Intra- and interoperator reliabilities of assessing upper trapezius stiffness at 0° of cervical flexion. (c, d) Intra- and interoperator reliabilities of assessing upper trapezius stiffness at 50° of cervical flexion (the continuous lines represent the mean difference, while the dotted lines show the 95% upper and lower limits of agreement).

Bland and Altman plots of intra- and interoperator reliabilities with 0° of cervical flexion are shown in Figures 3(a) and 3(b). The mean difference was -0.57 or 4.16 kPa, and the 95% limits of agreement were -12.5 to 11.3 kPa or -3.07 to 4.16 kPa. Other plots of intra- and interoperator reliabilities at 50° of cervical flexion are shown in Figures 3(c) and 3(d). The mean difference was 1.29 or 0.37 kPa and the 95% limits of agreement were -21.1 to 23.7 kPa or -14.7 to 15.4 kPa.

### 3.3. Changes in Upper Trapezius Stiffness during Cervical Flexion

**Figure 4:**
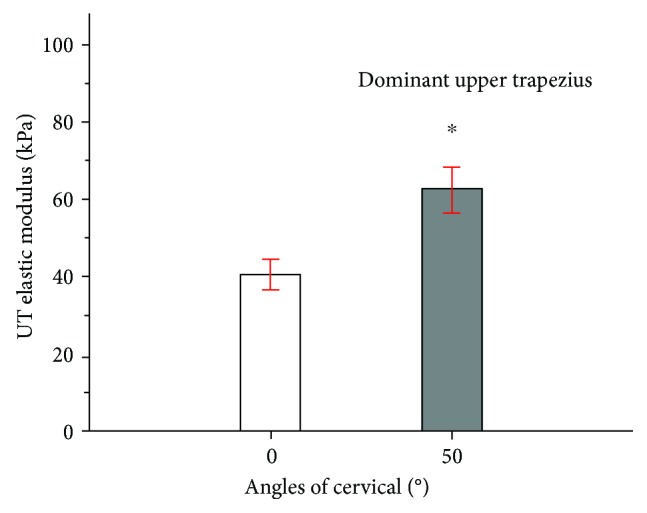
Mean and standard deviation of upper trapezius shear modulus examined during 0° (white bar) or 50° (gray bar) of cervical flexion. ^∗^Significant intergroup difference (*P* < 0.05).

The mean upper trapezius stiffness value was 40.47 kPa at 0° of cervical flexion. By comparison, the stiffness was 60.83 kPa at 50° of cervical flexion (*P* ≤ 0.001), with an increase of 35.58% (Figure 4).

### 3.4. Differences of Upper Trapezius Stiffness between the Dominant and Nondominant Sides

**Figure 5:**
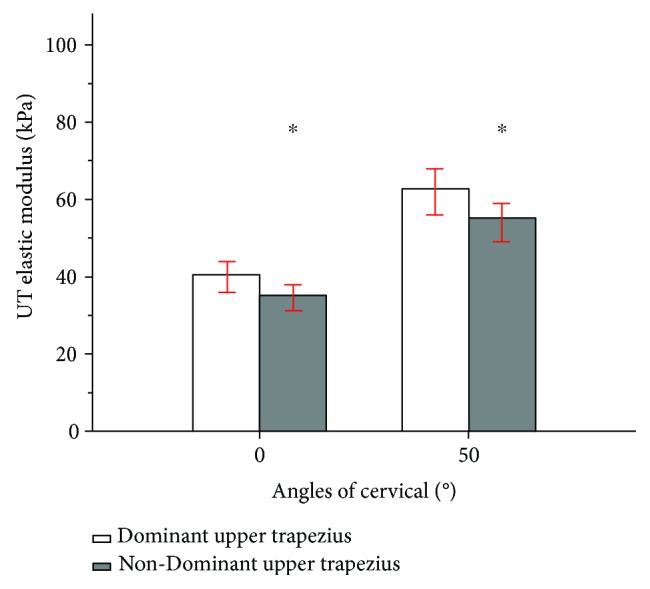
Mean and standard deviation in upper trapezius shear modulus examined between the dominant (white bar) and nondominant (gray bar) sides during 0° and 50° of cervical flexion. ^∗^Significant intergroup difference (*P* < 0.05).

At 0° of cervical flexion, there was a significant difference in the elastic modulus of the upper trapezius between the dominant (40.47 kPa) and nondominant (35.25 kPa) sides (*P* ≤ 0.001). During 50° of cervical flexion, a significant difference in the elastic modulus of the upper trapezius muscle was found between the dominant (62.83 kPa) and nondominant (55.21 kPa) sides (*P* ≤ 0.001) (Figure 5).

## 4. Discussion

SWE is a feasible instrument for assessing the shear modulus of the upper trapezius at different cervical degrees. We noted excellent intra- and interoperator reliabilities for evaluating upper trapezius stiffness via SWE with relatively low SEM and MDC values. A significant difference was obtained in upper trapezius stiffness between the 0° and 50° of neck flexion. We also noted a significant difference in upper trapezius muscle stiffness between the dominant and nondominant sides.

### 4.1. Intra- and Interoperator Reliabilities

In the present study, intraoperator reliability was good for assessing the upper trapezius at 0° of cervical flexion (ICC = 0.86) and 50° of cervical flexion (ICC = 0.85), but the interoperator reliability at 0° of cervical flexion (ICC = 0.98) and 50° of cervical flexion (ICC = 0.94) was excellent. Our findings were relatively consistent with those of previous studies evaluating skeletal muscles. SWE was used to assess the upper trapezius with the arm at rest and at 30° of abduction [19]. The intraoperator (ICC = 0.87) and interoperator (ICC = 0.78) reliabilities were good with the arm at rest, corresponding to the SEM < 6.23 kPa and MDC < 17.26 kPa. One study revealed the same ICC values (ICC = 0.97) for intra- and interoperator reliabilities of assessing the upper trapezius using the Myoton PRO with the shoulder in a neutral position [23]. One possible reason for the higher ICC values for intraoperator reliability than those in our study may be related to the use of different instruments for assessing the upper trapezius. No study of the reliability of assessing the upper trapezius at 50° of neck flexion is available to enable a comparison. The intra- and interoperator reliabilities of assessing other skeletal muscles using SWE were excellent. All regions of supraspinatus muscle elasticity were quantified by SWE and showed satisfactory intra- and interobserver reliabilities: ICC = 0.945–0.970 or ICC = 0.882–0.948 [24]. Only intraoperator reliability (ICC ≥ 0.90) was used to evaluate the pectoralis minor muscle in six different positions [25]. To summarize, SWE is a highly repeatable technique for quantifying muscle elasticity.

In our study, the ICC values for interoperator reliability were relatively high compared to those of intraoperator reliability. Considering possible explanations for these differences, we considered that the 5-day interval from the first measurement might have been a dominant factor, as the amount of exercise and other external factors in this 5-day period may have influenced the experiment's accuracy. The ICC value for 0° of cervical flexion may have been superior to that of 50° of cervical flexion due to measurement errors. In comparison to keeping the cervical flexion at 0°, maintaining a cervical flexion of 50° according to the Goniometer Pro was difficult as holding the cervical flexion at relatively higher degrees of flexion accurately was difficult, and thus, the error of measuring the flexion angles in such cases cannot be ignored. The findings from this study have indicated that the SWE is a credible instrument for evaluating upper trapezius stiffness.

The Bland-Altman plots of our study data further verified the consistency of our findings. It is clinically acceptable to obtain measurement differences within the limits of agreement [26]. As seen in Figure 3, almost all of the data points were within the 95% confidence limit. Therefore, the consistency of our study data is satisfactory.

### 4.2. Alterations in Upper Trapezius Stiffness during Cervical Flexion

The shear elastic modulus of the upper trapezius muscle during cervical flexion was quantified using SWE in this study. Our results demonstrated that the mean shear modulus of the upper trapezius at 0° of cervical flexion was 40.47 kPa; this value increased to 62.83 kPa at 50° of cervical flexion. Also, the change in the shear modulus was greater than the MDC (7.04 kPa), indicating true changes. This is the first study to examine the effect of different neck positions on the elastic modulus of the upper trapezius muscle. It is difficult to directly compare our findings to those of previous studies. However, previous studies investigated the effect of various shoulder positions on the elastic modulus of the upper trapezius muscle. For example, using SWE, Leong et al. [19] reported that the elastic modulus of the upper trapezius muscle was influenced by shoulder abduction, specifying an increase of 55.23% of shear elastic modulus during arm positions of 0° to 30° of abduction. In addition, our recent study demonstrated that shoulder flexion could affect upper trapezius stiffness using a handheld Myoton PRO device. We also found a 14.2% increase in upper trapezius stiffness at 0° to 60° of shoulder flexion [23]. In addition, using SWE, Maher et al. [27] reported that the elastic modulus of the upper trapezius was affected by a posture change. Specifically, they reported a 12.2% increase with a change from a prone to a sitting position. In another study, an increase in upper trapezius activity was detected among smart phone users. The fatigue of the upper trapezius assessed by EMG during cervical flexion revealed a value of −0.2 ± 1.3 Hz at 0° of neck flexion that increased to −3.5 ± 5.6 Hz at 50° of neck flexion [11]. Here, we found an increase of 35.58% in the elastic modulus of the upper trapezius with a change from 0° to 50° of cervical flexion.

The upper trapezius, as a neck extensor muscle, maintains cervical spine stability. The external flexion torque caused by cervical flexion has a significant effect, directly increasing the extensor muscle load. Moreover, muscle overload in a poor posture can easily lead to pathological changes to the neck and shoulder, while upper trapezius stiffness can significantly increase among people with neck and shoulder disorders. For example, using SWE, Leong et al. [7] demonstrated a 20% stiffer upper trapezius among subjects with rotator cuff tendinopathy compared with that in healthy subjects. Ishikawa et al. [8] reported an increased upper trapezius stiffness among people with neck and shoulder complaints compared with healthy subjects. Therefore, the 35.58% increase in the shear modulus of the upper trapezius during cervical flexion noted in our study further verifies that poor posture may be a risk factor for neck and shoulder complaints.

Furthermore, recent studies suggested that many therapies could decrease upper trapezius stiffness. One study found that dry needling could be used to reduce the elastic modulus of the upper trapezius, reporting a 12.8% reduction in the elastic modulus of the upper trapezius pre- versus posttreatment [27]. Cervical traction is a good way to relieve neck discomfort. Sung-Yong and Jung-Hyun [28] examined the influence of three therapies (cervical traction, cranial rhythmic impulse, and McKenzie exercise) on upper trapezius stiffness using a Myoton PRO and reported that upper trapezius stiffness was significantly reduced by 5.5 N/m after cervical traction. Furthermore, massage therapy is also a good way to relieve pain and tension, as a 19.3% decrease in upper trapezius activity was measured by EMG after massage treatment [29].

The MDC was calculated to detect true changes. In our study, the elastic modulus of the upper trapezius should have been greater than 7.04 kPa with different operators to reveal the true changes with reassessed measurements.

### 4.3. Differences in Upper Trapezius Stiffness between the Dominant and Nondominant Sides

Here, we found a significant difference in the elastic modulus of the upper trapezius between the dominant and nondominant sides. The high utilization of the dominant versus the nondominant side may contribute to the long-term usage of the extensor muscles, which reasonably explains the differences in stiffness. Uthaikhup et al. [30] examined the thickness of the lower trapezius and found greater lower trapezius muscle thickness on the dominant versus the nondominant side of 0.43 ± 0.02 mm. Fatigue of the upper trapezius also differed bilaterally; the upper trapezius on the dominant side was less fatigable by surface EMG [31]. These results were similar to our findings, which might support the notion of long-term usage of the extensor muscles on the dominant side. However, another study reported no significant difference in muscle fatigue between the dominant and nondominant sides [11]. The different results might be caused by differences between studies, such as those associated with the participant sex, age, and work experience. Therefore, the stiffer upper trapezius on the dominant side might lead to a higher risk of neck and shoulder pain compared to the nondominant side. Our findings could be useful in the prevention of neck and shoulder pain, and we should consider the differences in the shear elastic moduli of the upper trapezius between the dominant and nondominant sides.

SWE, a new technique that provides relatively standard elastic parameters of biological tissues, has high accuracy and sensitivity, features good repeatability, and is a simple operative method that measures muscle elasticity from the initial qualitative assessment to the quantitative assessment. SWE has been widely used in healthy individuals for muscle assessments as well as in biomechanical studies [19, 32, 33]. Previous comparisons of SWE and a muscle hardness meter showed that the former more precisely evaluated neck and shoulder muscle stiffness [34]. Moreover, assessing alterations in supraspinatus stiffness after a margin convergence technique using SWE contributed to a deeper insight into the biomechanical effect on the repaired supraspinatus and provided a scientific and reasonable rehabilitation plan [35]. Thus, SWE is expected to be an effective measurement tool for quantitatively evaluating the musculoskeletal system under various physiological and pathological conditions.

### 4.4. Limitations

This study had some limitations. First, only male subjects were included; therefore, sex-based differences could not be evaluated. Muscle discomfort caused by a computer test was more pronounced in male subjects in a previous study [36]. Further studies of the biomechanical characteristics of the upper trapezius of females are required. Second, we measured only one site of the upper trapezius; however, this cannot reflect the entire upper trapezius. It will be worth exploring the differences in stiffness values in different parts of the upper trapezius in the future. Third, all recruited subjects had no neck or shoulder complaints; therefore, a subsequent experiment will focus on assessing modulations in upper trapezius stiffness using SWE for people who suffer from neck and shoulder pain.

## 5. Conclusions

SWE is a potential tool for assessing upper trapezius elasticity with satisfactory reliability. Further studies should investigate the biomechanical properties of the upper trapezius using SWE among people with neck and shoulder complaints.

## Figures and Tables

**Table 1:** Subjects' demographic information (*N* = 20 male subjects).

	Mean ± SD
Age (years)	23.1 ± 2.7
Weight (kg)	70.6 ± 9.9
Height (m)	1.74 ± 0.04
Body mass index (kg/m^2^)	23.4 ± 2.7
Weekly exercise hours	2.8 ± 2.3

SD, standard deviation.

**Table 2:** Intra- and interrater reliabilities of SWE for assessing upper trapezius muscle stiffness.

	Cervical flexion at 0°			Cervical flexion at 50°		
	Mean ± SD	SEM	MDC	Mean ± SD	SEM	MDC
Operator A in test 1	40.47 ± 11.37	2.54	7.04	62.83 ± 22.42	5.01	13.89
Operator A in test 2	39.90 ± 11.62	2.59	7.20	64.12 ± 19.58	4.37	12.13
Operator B	41.01 ± 12.07	2.69	7.48	63.20 ± 21.77	4.86	13.49
ICC^a^ (95% CI)	0.86 (0.69–0.94)			0.85 (0.67–0.94)		
ICC^b^ (95% CI)	0.98 (0.96–0.99)			0.94 (0.86–0.97)		

ICC, intraclass correlation coefficient; CI, confidence interval; SEM (kPa), standard error of measurement of kPa; MDC (kPa), minimal detectable change; SD (kPa), standard deviation of kPa; kPa, kilo Pascal. ^a^Intraoperator reliability; ^b^Interoperator reliability.

## Data Availability

All data included in this study are available upon request from the corresponding author.
